# Stratification of Group A Streptococcal Pharyngitis Children Using Unsupervised Learning

**DOI:** 10.7759/cureus.65461

**Published:** 2024-07-26

**Authors:** Yoshifumi Miyagi

**Affiliations:** 1 Department of Pediatrics, Haibara General Hospital, Shizuoka, JPN

**Keywords:** k-modes, pca, unsupervised learning, pharyngitis, group a streptococcus

## Abstract

Background and objectives

Group A Streptococcus (GAS) is the most frequent cause of bacterial pharyngitis, and it is advised to selectively use rapid antigen detection testing (RADT). Currently, the decision to perform this test is based on pediatricians' observations, but the criteria are not well-defined. Therefore, we utilized unsupervised learning to categorize patients based on the clinical manifestations of GAS pharyngitis. Our goal was to pinpoint the clinical symptoms that should prompt further examination and treatment in patients diagnosed with pharyngitis.

Methods

We analyzed categorical data from 305 RADT-positive patients aged three to 15 years using the K-modes clustering method. Each explanatory variable's relationship with cluster variables was statistically examined. Finally, we tested the differences between clusters for continuous variables statistically.

Results

The K-modes method categorized the cases into two clusters. Cluster 1 included older children with lymph node tenderness, while Cluster 2 consisted of younger children with cough and rhinorrhea.

Conclusion

Differentiating streptococcal pharyngitis from common cold or upper respiratory tract infection based on clinical symptoms alone is challenging, particularly in young patients. Future research should focus on identifying indicators that can aid in suspecting streptococcal infection in young patients.

## Introduction

Group A Streptococcus (GAS) is the leading cause of bacterial pharyngitis [1]. Proper diagnosis is essential because untreated GAS pharyngitis can result in severe complications [2,3]. Recommendations from the American Heart Association, the Infectious Disease Society of America, and the American Academy of Pediatrics suggest the selective use of rapid antigen detection tests (RADT) [4-6]. Although the McIsaac score assists in distinguishing GAS pharyngitis from clinical presentations in children, bacterial culture remains the gold standard for diagnosing GAS pharyngitis [7,8]. However, pharyngeal cultures require 24-48 hours and specialized equipment. To address these issues, RADT was developed, providing detection of GAS within minutes. Despite its advantages, the current decision to utilize the test is based on pediatricians' clinical assessments, and the specific criteria for testing remain unclear.

Unsupervised learning is a machine learning approach used to infer patterns from datasets containing unlabeled input data [9]. The primary objective of unsupervised learning methods is to identify similarities among data points and group similar data together [9]. Cluster analysis, a widely employed unsupervised learning technique, is commonly used in exploratory data analysis to uncover hidden patterns and organize data into groups [9]. Unsupervised learning can identify and categorize various features from data without the need for labeled inputs [9]. The K-modes method, known for its high accuracy in clustering categorical data by selecting attributes based on information gain [10], has been effectively applied in medical data analysis [11].

Indicators are needed to suggest when to test and initiate antimicrobial therapy in patients diagnosed with pharyngitis. Therefore, we used unsupervised learning to stratify patients based on the clinical presentation of GAS pharyngitis and to identify the characteristics of clinical symptoms that should guide the next examination and treatment in patients diagnosed with pharyngitis.

## Materials and methods

Data collection

Ethics committee approval was not required for this study because we used publicly available supplemental data from previously published articles. We used data from a French prospective multicenter cross-sectional study [12]. The study included 305 children, aged three to 15 years, with RADT (Rapid Immunochromatography Strip Assay, Streptatest; Dectra Pharm, Eckbolsheim, France) positive results, diagnosed with GAS pharyngitis between October 1, 2010, and May 31, 2011, who had either throat pain, tonsillar swelling, tonsillar exudate, or pharyngeal erythema.

Data preprocessing

The obtained data set included characteristics such as age, sudden onset sore throat, maximum body temperature (reported by the accompanying parent), sore throat, cough, rhinorrhea, conjunctivitis, headache, pharyngeal erythema, tonsillar swelling, tonsillar exudate, palatal petechiae, nausea and vomiting, abdominal pain, diarrhea, tender cervical lymph nodes, and the presence of scar-like skin patches. One outcome variable, the result of the RADT, was also extracted.

Data analysis

Seed was set to 42 for the analysis. Categorical data were extracted from all 305 patients and clustered using the K-modes method. This K-modes technique extends the K-means pattern for clustering categorical data by using simple match dissimilarity ratings or Hamming distances for categorical data and eliminating the limitations of K-means [10]. After one-hot encoding of the data, the shape of the data was checked using principal component analysis (PCA) [13,14]. PCA is a technique used to reduce the complexity of high-dimensional data while preserving its trends and patterns [15]. This is achieved by transforming the data into fewer dimensions, which effectively summarize the original features. PCA works by geometrically projecting data into a lower-dimensional space, known as principal components (PCs) [15]. The objective is to capture the essential information of the data using a limited number of PCs. The first principal component (PC1) is selected to minimize the total distance between the data points and their projection onto PC1, which simultaneously maximizes the variance of the projected points. Each subsequent PC is chosen to be uncorrelated with the previous ones and to maximize the remaining variance, ensuring that the PCs are geometrically orthogonal to each other. The appropriate number of clusters was set, and the distribution of PCA was checked for each explanatory variable. Additionally, the relationship between each explanatory variable and the cluster variables was statistically analyzed. For variables showing significant differences, the distribution of the number of cases was checked, and a cross table was created to evaluate differences in characteristics between clusters. Finally, differences between clusters were statistically tested for continuous variables.

Statistical analysis

The study utilized the Python programming language, specifically version 3.7.12. For statistical analysis, a Mann-Whitney U-test was employed to analyze continuous variables, while a Fisher's exact test was used for categorical variables.

## Results

**Figure 1:**
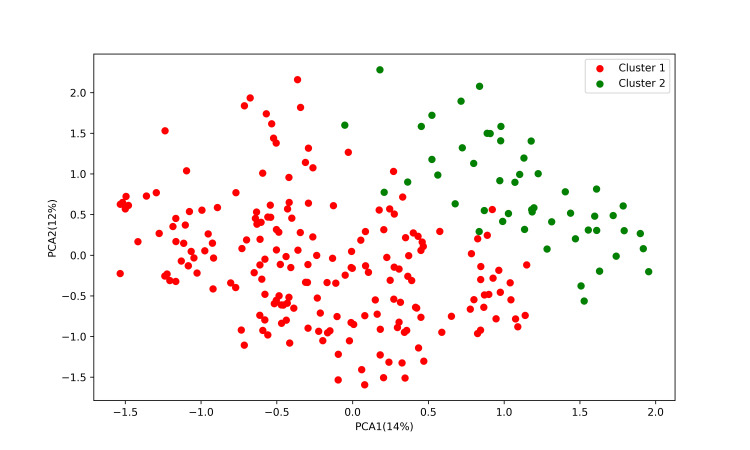
Principal component analysis of 305 children with streptococcal pharyngitis clustered by the K-modes method

Using the PCA scatterplot as a reference, the K-modes method was used to classify the cases into two clusters; the contribution of the two PCAs was 14% and 12%, respectively (Figure 1). The scatter plots of the PCA projected in two dimensions showed that each sample was divided into two large groups (Figure 1).

Cluster 1 included 247 cases, and Cluster 2 included 58 cases (Table 1). Of the 16 categorical variables, lymph node tenderness, cough and rhinorrhea were shown to be associated with the cluster (Table 1).

**Table 1:** Characteristics of this study divided into two clusters ¶　p-value < 0.05 was considered significant. Adj_p indicates false discovery rate corrected by multiple testing using the Benjamini-Hochberg method. IQR, Interquartile Range.

	Cluster 1		Cluster 2		p_value	Adj_p
	(N=247)		(N=58)			
Categorical variables						
	(+)	(-)	(+)	(-)		
throat pain	208	39	48	10	0.843	0.917
lymphadenopathy	165	82	34	24	0.284	0.510
lymph node tenderness	73	174	7	51	0.007	0.033^¶^
tonsillar swelling	187	60	37	21	0.071	0.255
tonsillar exudate	48	199	7	51	0.254	0.509
sudden onset	206	41	44	14	0.187	0.438
cough	36	211	58	0	<0.001	<0.001^¶^
rhinorrhea	43	211	58	0	<0.001	<0.001^¶^
conjunctivitis	3	244	3	55	0.085	0.255
headache	65	182	19	39	0.330	0.541
pharyngeal erythema	226	21	54	4	1.000	1.000
palatal petechiae	74	173	12	46	0.195	0.428
abdopain	80	167	17	41	0.755	0.917
diarrhea	6	241	3	55	0.379	0.570
nausea/vomit	60	187	15	43	0.867	0.917
scarlet	49	198	10	48	0.716	0.917
Continuous variables						
age (median[IQR))	5.0 [4.0, 7.0]		4.0 [4.0, 5.8]		<0.001	0.002^¶^
temperature (median[IQR))	38.0 [38.0, 39.0]		38.0 [38.0, 39.0]		0.816	0.916

**Figure 2:**
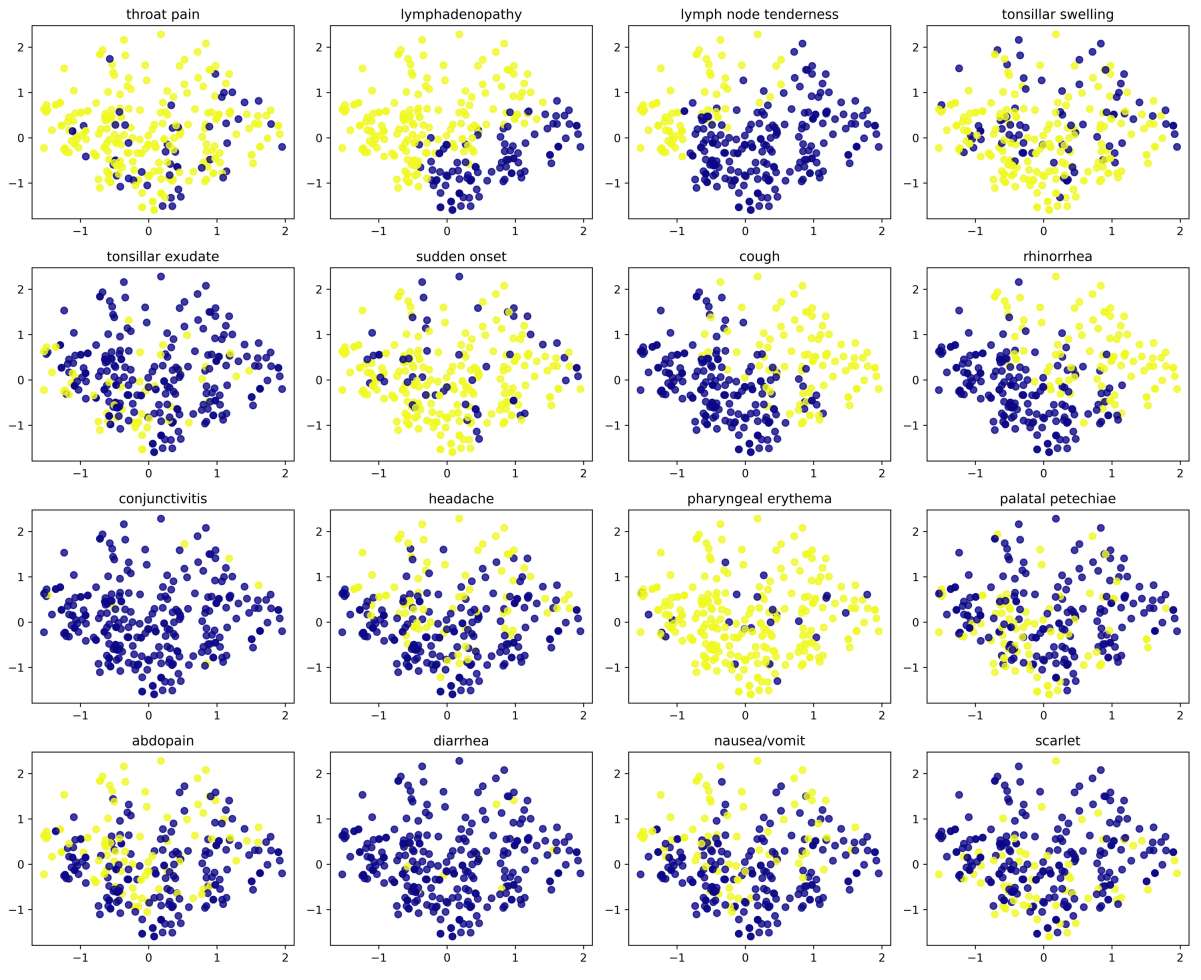
Principal component analysis for each of 305 children with streptococcal pharyngitis categorical data Yellow dots indicate symptomatic, blue dots indicate not symptomatic. The x-axis indicates the first principal component (14%) and the y-axis indicates the second principal component (12%). Values are principal component scores.

Scatter plots for the explanatory variables showed the same separation as for cluster classification in cough and rhinorrhea (Figure 2). More than half of the lymph node tenderness also showed a symptomatic yellow dot within Cluster 1 (Figure 2). The rest were distributed without any relationship to clusters, as seen in the scatter plots (Figure 2).

Statistical tests of the two clusters and each explanatory variable found significant differences in lymph node tenderness, cough, and rhinorrhea (Table 1). Lymph node tenderness was significantly elevated in Cluster 1, and the latter two were significantly elevated in Cluster 2. It should be noted that cough and rhinorrhea were found in all cases in Cluster 2, which was considered a cluster that could not be judged as a common cold, such as upper respiratory tract infection (Table 1).

Furthermore, statistical tests of clusters and continuous variables showed significant differences in age (Table 1). This indicates that Cluster 1 represents an older population with lymph node tenderness, while Cluster 2 represents a younger population with cough and rhinorrhea.

## Discussion

Categorical data were extracted from all 305 patients and clustered using the K-modes method with the number of clusters set to two. Cluster 1 (older group) represents lymph node tenderness, while Cluster 2 (younger group) represents cough and rhinorrhea. The following discussion is based on the limited literature available.

Unsupervised learning analysis of medical data can identify subgroups from disease groups without correct labels [16]. In particular, K-mode methods provide excellent accuracy for clustering categorical data when selecting attributes based on information gain [10] and have been used effectively in medical data [11].

Using machine learning, we previously reported that important clinical findings in pediatric patients with pharyngitis were palatal petechiae, scarlatiniform rash, tender cervical lymph nodes, and age [17]. Importantly, age was included, suggesting the need for stratification; group A streptococci cause pharyngitis, impetigo infectiosa, rheumatic fever, and acute glomerulonephritis in infants [18]. Group A streptococcal infections, particularly streptococcal toxic shock syndrome and necrotizing fasciitis, are potentially life-threatening and have a high mortality rate [19]. On the other hand, clinical findings suggestive of streptococcal infection in infancy are not evident except for the common pharyngitis findings.

In this study, cough and rhinorrhea were more prominent in the younger cluster of GAS pharyngitis in children aged three to 15 years, suggesting that it is very difficult to differentiate GAS pharyngitis from upper respiratory tract inflammation, including cold symptoms, by clinical findings. Additionally, the number of clusters with more prominent lymph node tenderness increased with age, suggesting that cervical palpation is an extremely important clinical examination finding. Currently, all patients with symptoms of pharyngitis in infancy should be tested, although there is concern that the sensitivity of RADT may be insufficient for this purpose [20]. New molecular tests based on nucleic acid amplification show higher accuracy and rapid results, but their cost, complexity, and very high sensitivity may limit their widespread use [20].

The strength of this study lies in its demonstration of the feasibility of using unsupervised learning to stratify GAS pharyngitis based on clinical findings. Additionally, the study successfully provided a noninvasive perspective by reusing previously published data. However, several limitations should be noted. Firstly, a positive RADT result does not definitively indicate pure GAS pharyngitis. Furthermore, the study did not include data for children under the age of three, thereby limiting the stratification of cases in the infant population.

## Conclusions

The analysis was based on the clinical presentation of 305 cases of streptococcal pharyngitis in children aged three to 15 years and was performed using unsupervised learning with the K-modes method. Subjects were divided into two subgroups: the group with common cold symptoms of cough and rhinorrhea had a lower rate of lymph node tenderness and were younger. It is difficult to distinguish streptococcal pharyngitis from the common cold or upper respiratory tract infection based on clinical symptoms in young patients. Future studies are needed to find indicators to suspect streptococcal infection, including in patients younger than three years.

## Appendices

Dataset used in this study: https://doi.org/10.1371/journal.pone.0172871.s002
